# Comparative retention and effectiveness of migraine preventive treatments: A nationwide registry‐based cohort study

**DOI:** 10.1111/ene.16062

**Published:** 2023-09-27

**Authors:** Marte H. Bjørk, Solveig Borkenhagen, Francisco Oteiza, Aud N. Dueland, Frank E. Sørgaard, Erik Magnus Sæther, Christoffer Bugge

**Affiliations:** ^1^ Department of Clinical Medicine University of Bergen Bergen Norway; ^2^ Department of Neurology Haukeland University Hospital Bergen Norway; ^3^ NorHEAD, Norwegian Headache Research Centre Norwegian University of Science and Technology Trondheim Norway; ^4^ Oslo Economics Oslo Norway; ^5^ Sandvika Nevrosenter Sandvika Norway; ^6^ Department of Neurology Oslo University Hospital Oslo Norway; ^7^ Novartis Norge AS Oslo Norway; ^8^ Department of Health Management and Health Economics University of Oslo Oslo Norway

**Keywords:** botulinum toxin, CGRP antibodies, headache, prophylaxis, triptan

## Abstract

**Background and purpose:**

Little is known about the comparative effects of migraine preventive drugs. We aimed to estimate treatment retention and effectiveness of migraine preventive drugs in a nationwide registry‐based cohort study in Norway between 2010 and 2020.

**Methods:**

We assessed retention, defined as the number of uninterrupted treatment days, and effectiveness, defined as the reduction in filled triptan prescriptions during four 90‐day periods after the first preventive prescription, compared to a 90‐day baseline period. We compared retention and efficacy for different drugs against beta blockers. Comparative retention was estimated with hazard ratios (HRs), adjusted for covariates, using Cox regression, and effectiveness as odds ratios (ORs) using logistic regression, with propensity‐weighted adjustment for covariates.

**Results:**

We identified 104,072 migraine patients, 81,890 of whom were female (78.69%) and whose mean (standard deviation) age was 44.60 (15.61) years. Compared to beta blockers, botulinum toxin (HR 0.43, 95% confidence interval [CI] 0.42–0.44) and calcitonin gene‐related peptide pathway antibodies (CGRPabs; HR 0.63, 95% CI 0.59–0.66) were the least likely to be discontinued, while clonidine (HR 2.95, 95% CI 2.88–3.02) and topiramate (HR 1.34, 95% CI 1.31–1.37) were the most likely to be discontinued. Patients on simvastatin, CGRPabs, and amitriptyline were more likely to achieve a clinically significant reduction in triptan use during the first 90 days of treatment, with propensity score‐adjusted ORs of 1.28 (95% CI 1.19–1.38), 1.23 (95% CI 0.79–1.90), and 1.13 (95% CI 1.08–1.17), respectively.

**Conclusions:**

We found a favorable effect of CGRPabs, amitriptyline, and simvastatin compared with beta blockers, while topiramate and clonidine were associated with poorer outcomes.

## INTRODUCTION

Migraine is a headache disorder characterized by attacks of pulsating unilateral headache, accompanied by nausea, and/or photo‐ and phonophobia, and exacerbated by physical exertion [1]. The global 1‐year prevalence of migraine is 18% in women and 9% in men [2], with 959 million people experiencing migraine worldwide [3]. In 2019, migraine was second among the causes of disability and the most important cause of disability among women under 50 years [2, 3, 4, 5]. Patients with chronic migraine have at least 15 headache days a month, with at least 8 days of having headaches with migraine features [1]. Migraine preventive drugs reduce migraine attack frequency, intensity, duration, and disability [6]. Even though almost 40% of all patients with migraine and most patients with chronic migraine would benefit from migraine preventive drugs, only 3%–13% use such drugs and adherence has been found to be low [7, 8, 9, 10, 11, 12, 13].

Recently, many new treatments for migraine have become available. The introduction of botulinum toxin A (BtA) for chronic migraine and drugs specifically designed for migraine prevention, such as the calcitonin gene‐related peptide pathway antibodies (CGRPabs), have led to a need for a better understanding of how patients are affected by different treatment options in a real‐world setting. Little is known about the comparative effectiveness and tolerability of migraine preventive drugs, and this limits access to some of the treatments [7, 14].

Prescription data provide opportunities to study the treatment duration and effectiveness of migraine preventive drugs in a real‐world setting. Treatment duration can be analyzed by studying the time from treatment initiation to treatment discontinuation [15, 16, 17]. The uninterrupted use of preventive drugs suggest a satisfactory patient experience. Effectiveness can be approximated by measuring a patient's reduction in triptan prescriptions as an indication of improvement in migraine symptoms [18].

## METHODS

### Data source, design, and study cohort

We conducted a registry‐based cohort study of eligible migraine patients in Norway filling prescriptions for migraine preventive drugs between 2010 and 2020, using data from the Norwegian Prescription Database. This database contains detailed information on all prescriptions filled in pharmacies nationwide. Over‐the‐counter drugs and medication used in hospitals and nursing homes were not included. Drug expenses are universally covered in Norway, but reimbursement may require fulfillment of clinical criteria.

We defined patients with migraine as a person that had at least one prescription reimbursed for migraine (code G43 according to the 10th revision of International Statistical Classification of Diseases [ICD‐10]) or code N89 according to the International Classification of Primary Care (ICPC‐2) [19, 20, 21]. As there was imperfect compliance with the registration of reimbursement codes in the early years of the registry and triptans are almost exclusively used for the treatment of migraines in Norway, we also included patients with at least two prescriptions of triptans (Anatomical Therapeutic Chemical [ATC] classification code N02CC) [22] between 1 January 2008 and 31 December 2020 (*n* = 346,154 patients). To further ensure that the study population was composed exclusively of migraine patients, we excluded patients who had at any time had a drug reimbursed for cluster headache (ICD‐10: G44.0‐G44.2 or ICPC‐2 N90). We also excluded patients aged <2 years or >99 years at the time of their first prescription (*n* = 18,250; Figure S1). The dataset included all prescriptions filled by these patients (*n* = 54,246,980 prescriptions). Each prescription included the date of redemption, county of residence, year and month of birth and death, sex, reimbursement code (ICD‐10/ICPC2), pack size, drug strength, drug name, defined daily dose (DDD) and ATC code [22, 23].

Patients with no migraine preventive drug prescriptions between 1 January 2010 and 31 December 2019 were excluded (*n* = 223,832) to ensure at least 1 year of follow‐up. Patients with no triptan prescriptions were included in the analysis of retention but excluded from the analysis of effectiveness (Figure S1).

We defined a minimum effective dose for prophylactic use of migraine preventive drugs based on doses recommended to treat migraine in Norway (Table S1). Individual treatment periods were constructed based on the amount of DDDs retrieved and the minimum effective dose for each drug and defined as an uninterrupted period during which a patient retrieved enough DDDs of a specific drug to cover its minimum effective dose. Since these were based on minimum effective doses, no grace period between treatment periods was included. The analysis was restricted to each patient's first treatment period with each different migraine preventive drug type.

### Outcome variables

The primary outcomes were retention rate and 30% effectiveness for the following migraine preventive drugs groups: beta blockers (propranolol and metoprolol), candesartan, lisinopril, topiramate, CGRPabs (erenumab, fremanezumab, galcanezumab), BtA, clonidine, and simvastatin (Table S1). These drugs are recommended for migraine prophylaxis by clinical guidelines and/or are in common use for this indication in Norway [6, 24, 25, 26, 27, 28, 29]. As reimbursement practice has varied in Norway over the last 10 years, all prescriptions were included regardless of reimbursement code.

Retention rates were defined as the duration, in days, of uninterrupted treatment periods. Based on guidelines from the International Headache Society, we defined effectiveness as the reduction in a patient's triptan use over the course of 360 days after initiation of a migraine preventive drug, divided into 90‐day periods, compared to their triptan use during a 90‐day baseline period before they started the preventive drug (Table S2) [30]. The primary outcome was the percentage of patients achieving at least a 30% reduction in the use of triptans during the first 90 days since start of treatment. In each successive 90‐day period, only patients who had not discontinued treatment were included in the calculation of effectiveness.

Secondary outcomes were the proportion of patients achieving at least a 50% reduction in triptan use and the absolute change in triptan use measured in DDDs as a proxy for migraine days [30].

### Covariates

Covariates included were patient age, county of residence, selected comorbidities (acute myocardial infarction, congestive heart failure, hypertension, heart rhythm disorders, renal disease, depression, anxiety disorder, nicotine replacement products, epilepsy, mood disorders, type 2 diabetes and asthma), year of treatment start, previous or simultaneous use of other migraine preventive drugs, and frequent triptan use, defined as filling prescriptions for more than 16 DDDs within a 30‐day period (Table S3).

### Analyses

Beta blockers were selected as the active comparator for retention and effectiveness estimates since they are the most prescribed migraine preventive drugs in Norway and their effectiveness for this indication is well documented. Retention was estimated using the Kaplan–Meier estimator [31]. We compared retention for the different drugs using Cox regression adjusted for covariates to estimate hazard ratios (HRs).

Effectiveness was estimated using logistic regression to produce odds ratios (ORs) adjusted for covariates. To further account for differences in patient characteristics across drug types, we applied propensity‐score weighting (Supplementary Methods) [32]. Each logistic regression was restricted to patients in the migraine preventive drug group and beta blockers with overlapping propensity scores, resulting in covariate balance across groups (Figure S2).

All analyses were performed using STATA 17.

### Sensitivity analyses

In Norway, BtA and CGRPabs are only reimbursed for chronic migraine. Thus, subgroup analyses were performed for patients filling prescriptions for 16 or more and 15 or fewer triptan DDDs within 30 days before initiating migraine preventive drugs (high‐frequency and low‐frequency triptan users). Since patients are recommended 1–2 triptan DDDs per day, 16 DDDs indicate 8–16 migraine days/month, suggesting chronic migraine and/or medication overuse headaches [1].

We restricted the sample to migraine preventive medications prescribed with a migraine reimbursement code, to check whether our results were affected by the indication for use. To avoid confounding outcomes of effectiveness resulting from use of multiple drugs with preventive properties, we limited the study group to patients on migraine preventive drug monotherapy.

Patients using triptans before experiencing a cerebrovascular or cardiovascular event could be prescribed antihypertensive or statin treatment after said event and thus be advised no longer to use triptans [33]. Thus, we excluded patients with prescriptions of platelet aggregation antibodies or antithrombotic agents (ATC codes: B01AA, B01AC, B01AE, and B01AF).

Separate analyses of effectiveness were performed by extending the baseline period for BtA by 270 days as this treatment in Norway is sometimes initiated in hospital outpatient clinics, which is not captured in prescription data.

### Ethical approval

Ethical approval and a waiver of informed consent was granted by the Regional Committees for Medical and Health Research Ethics (Ref: 242126).

## RESULTS

The study population consisted of 104,072 patients (Figure S1). The mean (standard deviation [SD]) patient age at the time of their first treatment period was 44.60 (15.61) years and 78.69% (81,890) were female. Beta blockers (51,385 patients), candesartan (35,736 patients), and amitriptyline (34,504 patients) were the most prescribed drugs with migraine preventive properties.

### Retention

**FIGURE 1:**
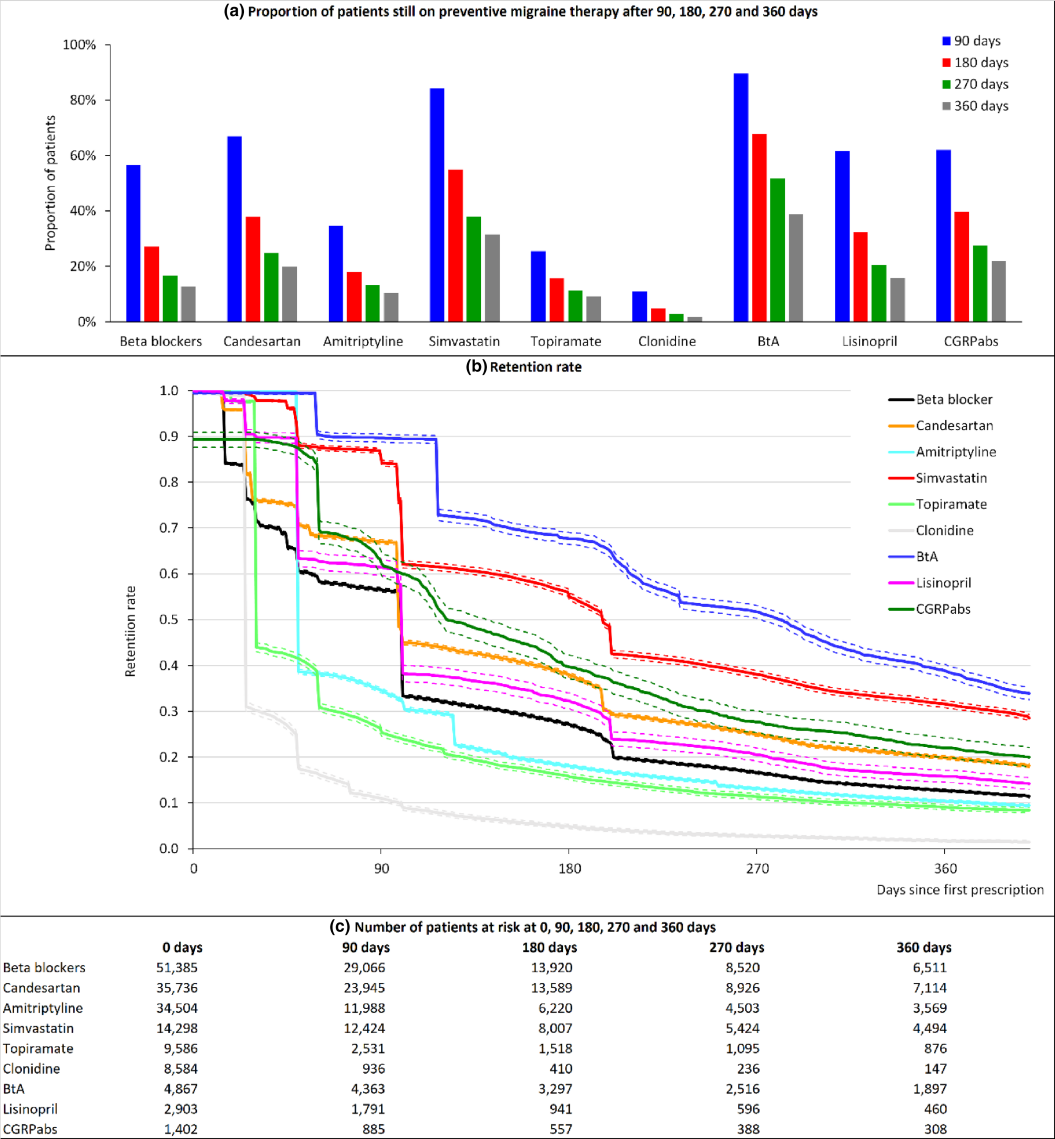
Duration of migraine preventive treatment by drug group. (a) Proportion of patients still on preventive migraine therapy after 90, 180, 270, and 360 days and proportion of patients still on each drug since first prescription (retention rate). (b) Retention rate with 95% confidence intervals (dotted lines) adjusted for patient characteristics, comorbidities, year of treatment start, previous use of migraine preventive drugs, and amount of triptan defined daily doses (DDDs) prescribed per month (above or below 16 DDDs/30 days in baseline period. BtA, botulinum toxin A; CGRPabs, calcitonin gene‐related peptide pathway antibodies. (c) Number of patients at risk at 0, 90, 180, 270 and 360 days from (b).

Retention rates varied substantially across the drug groups (Figure 1 and Table 1). BtA, simvastatin, and CGRPabs showed the highest proportion of patients on treatment 1 year after the first prescription, with 38.98% (1897/4867), 31.43% (4494/14,298), and 21.97% of patients receiving these drugs (308/1402), respectively. The proportion was lowest for clonidine (1.71%, 147/8584), topiramate (9.14%, 876/9586), and amitriptyline (10.34%, 3569/34,504).

**TABLE 1:** Patient characteristics, treatment length and probability of discontinuation by drug group.

	Beta blockers (reference)	Candesartan	Amitriptyline	Simvastatin	Topiramate	Clonidine	BtA	Lisinopril	CGRPabs	All
*Summary statistics*										
Number of treatment periods	51,385	35,736	34,504	14,298	9586	8584	4868	2903	1402	163,266
Number of patients	51,385	35,736	34,504	14,298	9586	8584	4868	2903	1402	104,072
Age^a^, years	44.74	44.46	42.96	57.77	38.78	45.80	41.99	50.62	44.02	44.60
SD	16.48	14.98	13.77	11.79	13.13	14.82	12.57	14.11	12.95	15.61
Gender (female)^a^, %	78.23	78.78	84.22	72.30	84.74	78.80	88.75	74.41	85.73	78.69
*N*	40,385	28,153	29,058	10,337	8123	6764	4320	2160	1202	81,890
Year of prescription	2014.35	2014.52	2014.71	2012.44	2015.55	2014.34	2016.92	2013.13	2018.74	2014.46
SD	3.18	3.16	3.05	2.78	3.00	3.01	1.68	3.07	0.44	3.17
High‐frequency triptan use^b^, %	45.75	57.43	50.66	34.04	70.43	34.39	81.66	51.09	95.08	50.76
*N*	23,511	20,524	17,479	4867	6751	2952	3975	1483	1333	82,875
Number of other MPDs earlier	0.80	0.89	0.89	1.27	1.62	1.06	2.23	1.40	2.93	1.02
SD	0.89	0.91	0.96	0.92	1.03	1.04	1.12	1.14	1.24	1.01
Monotherapy^b^, %	78.70	77.89	80.27	86.23	62.30	80.00	67.40	75.44	77.10	78.24
*N*	40,442	27,835	27,696	12,329	5972	6909	3281	2190	1081	127,735
Comorbidities (number)	0.90	1.03	0.80	1.30	0.84	1.10	0.82	1.36	0.89	0.96
SD	1.14	1.03	1.06	1.24	1.03	1.30	1.06	1.10	1.02	1.12
Baseline DDD triptans	15.58	20.19	15.89	12.50	28.83	11.79	33.92	21.90	55.50	17.97
SD	34.10	37.27	35.15	32.29	53.10	35.40	57.34	74.17	73.55	39.13
*Duration on treatment*										
Mean treatment length^c^, days	246.24	362.00	201.52	468.37	162.37	55.65	476.02	293.47	263.92	279.31
SD	2.72	4.25	2.94	6.26	5.12	1.24	9.20	11.74	9.50	1.69
Hazard ratio	–	0.76	1.03	0.71	1.34	2.95	0.43	0.94	0.63	–
(95% CI)	–	(0.75–0.77)	(1.02–1.05)	(0.69–0.72)	(1.31–1.37)	(2.88–3.02)	(0.42–0.44)	(0.91–0.98)	(0.59–0.66)	–

*Note*: Hazard ratios below 1 indicate a lower probability of treatment discontinuation. Patients on beta blockers were used as the (active) comparator in the estimation of these hazard rates.

Abbreviations: BtA, botulinum toxin A; CI, confidence interval; CGRPabs, calcitonin gene‐related peptide pathway antibodies; DDD, defined daily dose; MPD, migraine preventive drug; SD, standard deviation.

^a^ Only first treatment period per patient was evaluated in the calculation of age and gender; percentage was calculated based on number of patients.

^b^ Percentage was calculated based on number of treatment periods.

^c^ Restricted mean, largest observed analysis time was censored, and mean was underestimated. Treatment periods were defined as the uninterrupted number of days for which a patient retrieved enough DDDs to cover the minimum effective dose for a given drug.

Compared to beta blockers, the probability of treatment discontinuation adjusted for differences in covariates was lower for BtA (HR 0.43, 95% confidence interval [CI] 0.42–0.44), CGRPabs (HR 0.63, 95% CI 0.59–0.66), simvastatin (HR 0.71, 95% CI 0.69–0.72), and candesartan (HR 0.76,95% CI 0.75–0.77). Adherence rates for clonidine (HR 2.95, CI 2.88–3.02), topiramate (HR 1.34, 95% CI 1.31–1.37), and amitriptyline (HR 1.03, 95% CI 1.02–1.05) were lower than for beta blockers.

### Effectiveness

For all drugs combined, 50.22% of patients (40,912/81,472) achieved a 30% or greater reduction in triptan use during the 90 days following their first prescription, while 57.06% (25,654/44,963), 58.25% (14,887/25,557), and 59.45% (10,222/17,194) achieved this in the following observation periods during the first year.

**FIGURE 2:**
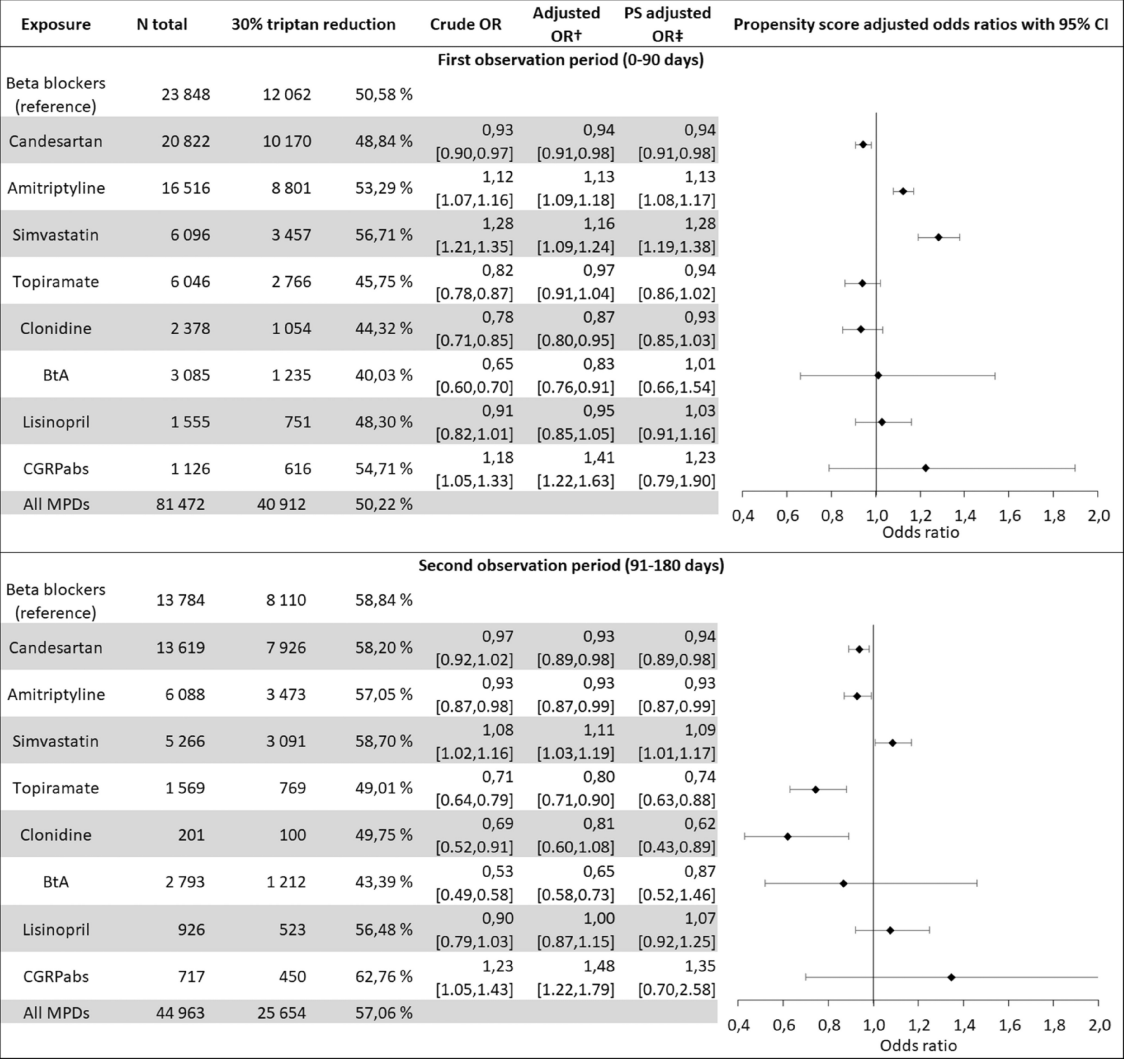
Relative effectiveness by drug group and observation period. Odds ratios (ORs) below 1 indicate a lower probability of effect (30% reduction in triptan use). ^†^Adjusted for covariates (patients age, county of residence at treatment start, number of relevant comorbidities, year of treatment start, previous or simultaneous use of migraine preventive drugs, and amount of triptan defined daily doses (DDDs) prescribed per month (above or below 16 DDDs within a 30‐day period). ^‡^Propensity score adjusted. BtA, botulinum toxin A; CI, confidence interval; CGRPabs, calcitonin gene‐related peptide pathway antibodies; MPD, migraine preventive drug.

During the first 90 days, the proportion with a 30% or greater reduction was largest for simvastatin (56.71%, 3457/6096), CGRPabs (54.71%, 616/1126) and amitriptyline (53.29%, 8801/16,516), and patients filling prescriptions for these drugs were more likely to achieve 30% triptan reduction than patients filling prescriptions for beta blockers, adjusted for covariates (Figure 2). Restricting the population to patients with overlapping propensity scores, the ORs for achieving a 30% triptan reduction were 1.28 (95% CI 1.19–1.38) for simvastatin, 1.23 (95% CI 0.79–1.90) for CGRPabs, and 1.13 (95% CI 1.08–1.17) for amitriptyline. These ORs were 0.94 (95% CI 0.91–0.98) for candesartan, 0.94 (95% CI 0.86–1.02) for topiramate, 0.93 (95% CI 0.85–1.03) for clonidine, 1.01 (95% CI 0.66–1.54) for BtA and 1.03 (95% CIs 0.91–1.16) for lisinopril.

**FIGURE 3:**
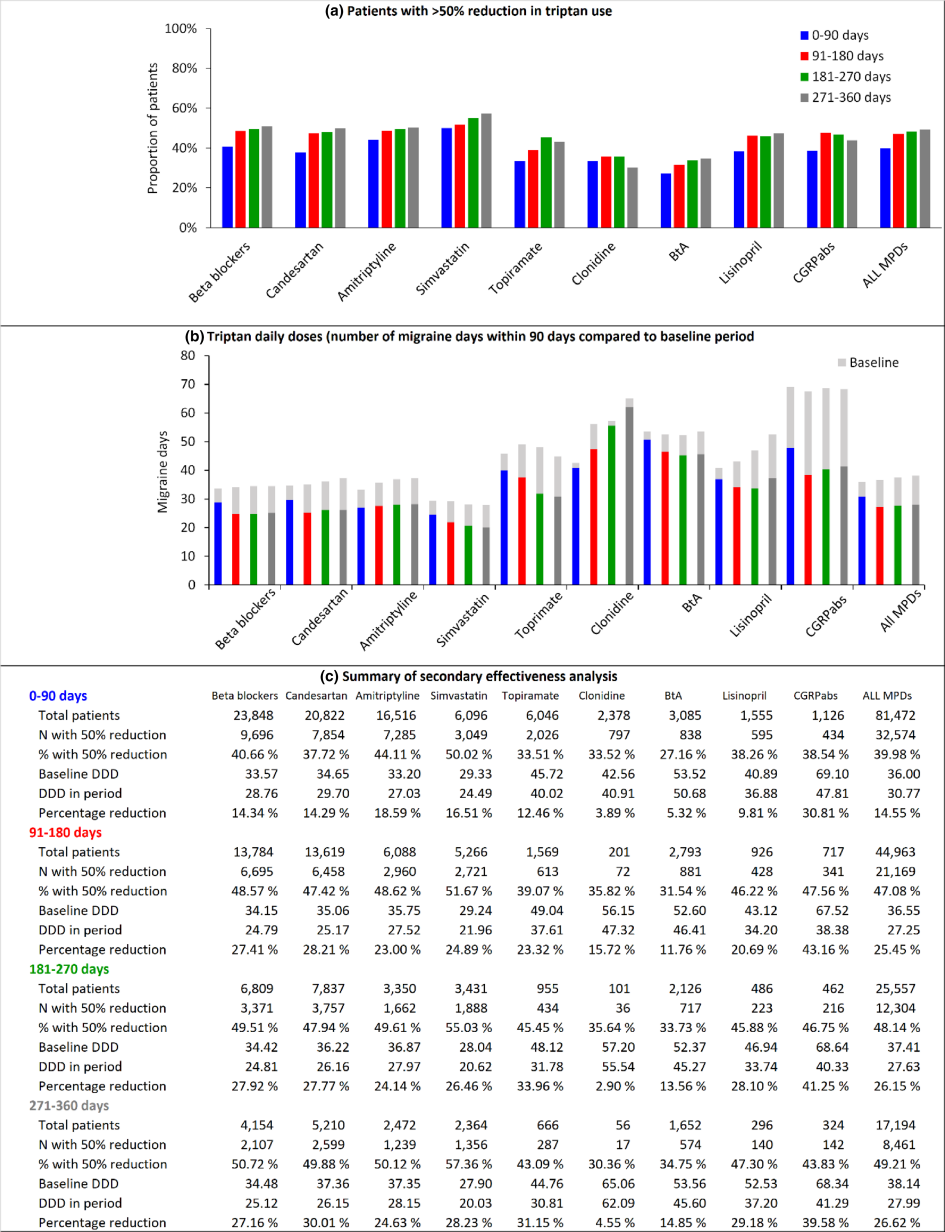
Reductions in triptan use by drug group and observation period. Proportion of patients achieving at least a 50% reduction in triptan use compared to their baseline levels (a), and (b) triptan daily doses used by patients before (light gray bars) and after starting treatment with migraine preventive drugs (colored bars). (c) summarize number of patiens, patients with 50% reduction, and change in DDD for each drug group and observation period. Baseline triptan consumption may vary across periods (within the same drug group), reflecting that the sample of patients who remain on treatment over each successive observation period is different. BtA, botulinum toxin A; CI, confidence interval; CGRPabs, calcitonin gene‐related peptide pathway antibodies; DDD, defined daily dose; MPD, migraine preventive drug.

During the first 90 days after their first migraine preventive prescription, 39.98% of patients (32,574/81,472) achieved a 50% reduction in triptan prescriptions for the whole sample, and 49.21% (8461/17,194) achieved this by the latest observation period. The pattern of effectiveness for a 50% reduction in triptan use for the different drugs was similar to that for the 30% reduction outcome (Figure 3).

The mean reduction in daily triptan doses during the first 90 days of treatment was 14.55% (5.23/36.00) for all migraine preventive drugs combined. The mean reduction was 30.81% (21.29/69.10) for CGRPabs, 18.59% (6.17/33.20) for amitriptyline, and 16.51% (4.84/29.33) for simvastatin. The other drugs were associated with a reduction of 14.34% or less (Figure 3). For patients staying on the drug after 90 days, the mean triptan reduction over the course of the remaining first year of treatment ranged from 39.58% (27.05/68.34) to 43.16% (29.14/67.52) for CGRPabs, 23.32% (11.44/49.04) to 33.96% (16.34/48.12) for topiramate, 27.77% (10.06/36.22) to 30.01% (11.21/37.36) for candesartan, 27.16% (9.37/34.48) to 27.92% (9.61/34.42) for beta blockers, 20.69% (8.92/43.12) to 29.18% (15.33/52.53) for lisinopril, 24.89% (7.28/29.24) to 28.23% (7.88/27.90) for simvastatin, 11.76% (6.18/52.60) to 14.85% (7.95/53.56) for BtA, and 2.90% (1.66/57.20) to 15.72% (8.82/56.15) for clonidine.

### Sensitivity analyses

**FIGURE 4:**
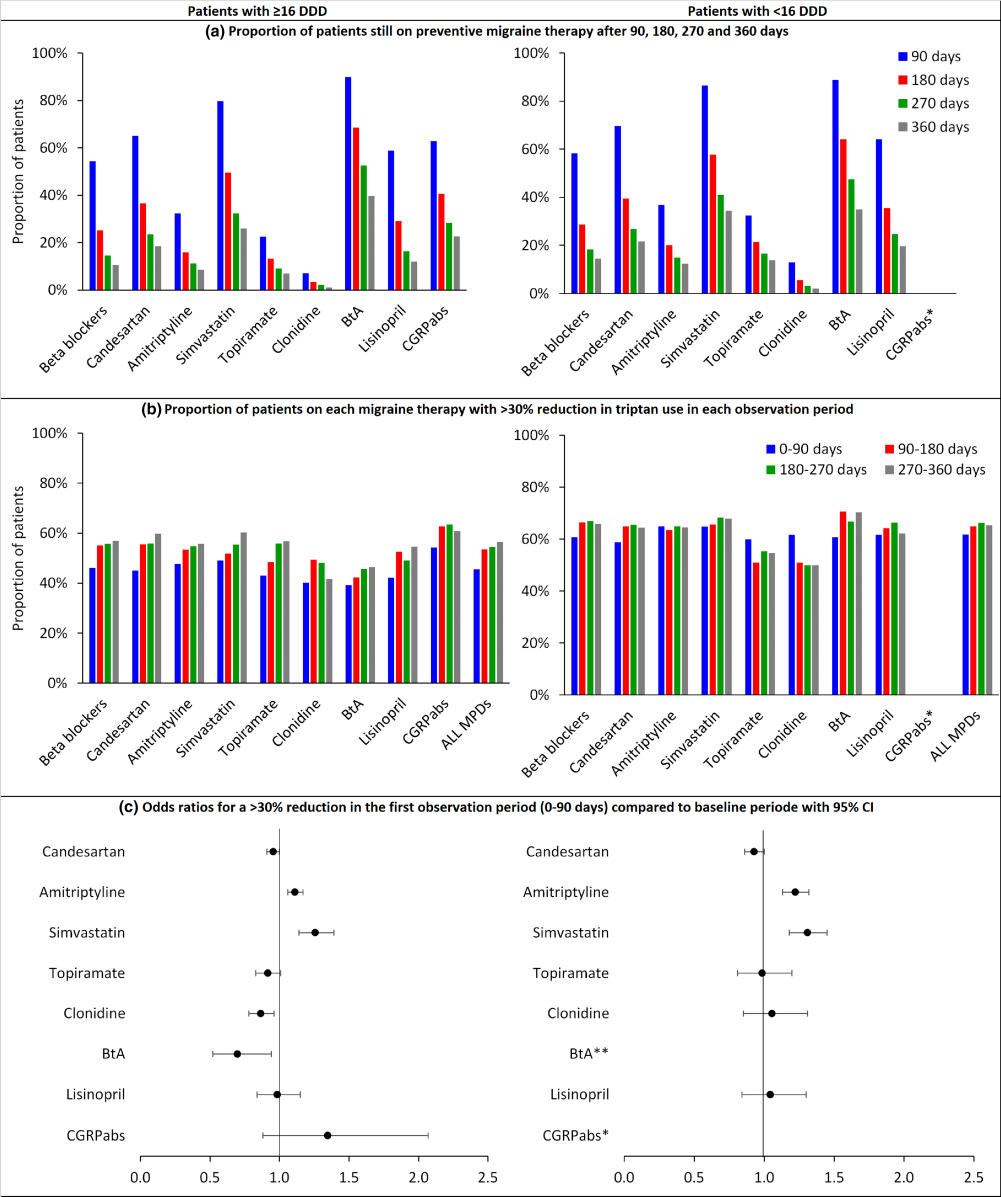
Sensitivity analysis: retention and effectiveness for patients stratified by triptan frequency (defined daily dose [DDD]). Retention (a) and effectiveness (b, c) for patients with a DDD above and below 16, by migraine preventive drug (MPD) group *Calcitonin gene‐related peptide pathway antibodies (CGRPabs) were excluded from the analysis of patients with <16 DDDs due to small sample size. **Botulinum toxin A (BtA) was excluded due to few observations in the propensity score‐adjusted analyses for patients with <16 DDDs. CI, confidence interval.

The patterns of retention and effectiveness were similar when subdividing the patient population into high‐frequency and low‐frequency triptan users. Overall, the proportion of patients achieving 30% reduction in triptan prescriptions was lower among the high‐frequency group (45.58% [26,457/58,050] vs. 61.72% [14,455/23,422]; Figure 4 and Table S4). The results from the propensity score‐adjusted comparisons with beta blockers were similar to those from the whole study population but with larger CIs, except for the BtA group (Figure 4, lower panel). Restricted to the patient group using most triptans, the BtA group had statistically significantly lower odds than the beta blocker group for achieving a 30% triptan reduction (OR 0.70, 95% CI 0.52–0.94; Figure 4).

The results for BtA after shifting the baseline period did not change much (Figure S3). Similarly, restricting the analysis to patients with a migraine reimbursement code, patients on monotherapy, or patients without prescriptions of platelet aggregation antibodies or antithrombotic agents did not influence the results much (Figure S4).

## DISCUSSION

To our knowledge, only one study has compared CGRPabs directly to another migraine preventive drug [34]. In that study, erenumab had better tolerability than topiramate and was more effective in an intention‐to‐treat analysis [35, 36, 37]. In our study including all patients prescribed CGRPabs in Norway during the study period, CGRPabs were associated with higher retention, supporting these data. Their effectiveness, measured as reduction in triptan use, was also higher than beta blockers. However, baseline triptan use was considerably higher for patients on CGRPabs. When restricting the analysis to patients with similar background characteristics (including triptan use), no statistically significant difference in effectiveness remained between the CGRPabs and beta blockers.

Simvastatin was associated with higher effectiveness and retention than beta blockers. Previous randomized controlled studies have shown that simvastatin and atorvastatin can prevent migraine attacks with similar response rates to those of other preventive drugs [38, 39, 40, 41]. As most of these studies were recently published, many patients in our study probably used simvastatin to prevent vascular incidents and not for their migraine, and this could have influenced the results [38, 41].

Amitriptyline use was associated with lower retention than beta blockers but higher effectiveness during the first 90 days of treatment. Although frequently used and recommended for migraine treatment, few studies have previously evaluated its effectiveness [42].

In our analyses, BtA was associated with the greatest treatment retention of all the drugs. However, the proportion of patients with reduced triptan use was lower for BtA than for beta blockers. Placebo‐controlled studies of BtA also indicate that its effects are greater in terms of reduction of headache days than in terms of acute medication intake [43, 44]. We did not estimate potential reduction in non‐specific acute medication (nonsteroidal anti‐inflammatory drugs, paracetamol, opioids etc.) and did not have access to over‐the‐counter drugs or drugs obtained without a prescription over the internet.

Antihypertensive agents (candesartan, clonidine and lisinopril) showed effectiveness as beta blockers, in line with a randomized controlled study of candesartan versus propranolol [45]. However, candesartan was associated with higher levels of retention. Clonidine, deemed possibly effective in the American Headache Society Position Statement on Migraine Treatments in 2018, was associated with significantly poorer retention and effectiveness than beta blockers [7, 46].

Topiramate was also associated with low levels of retention and effectiveness compared to beta blockers. Low tolerability for topiramate is well known from previous studies [42].

A strength of this study is that it includes all migraine patients filling prescriptions for preventive migraine treatment in Norway between 2010 and 2020. Retention and effectiveness could thereby be studied in the complete patient population without selection bias or loss of follow‐up. The selection of patients into clinical trials and consent‐based registries may limit external generalizability [42]. Knowledge about the overall tolerability and effectiveness for different drugs and drug classes in unselected populations from real‐world studies can inform doctors, patients and other stakeholders when considering different treatment options.

We adjusted for a range of possible confounders influencing the choice of migraine preventive drugs, including comorbidities and the number of previously failed migraine preventive drugs. We further accounted for patient characteristics in different drug groups by using propensity score‐weighted analyses. We nevertheless cannot rule out that unmeasured confounding factors impacted the results when comparing estimates to beta blockers. Finally, we conducted a range of sensitivity analyses that showed that our findings were robust to different sample specifications.

However, relying on prescription data has important limitations. We tested effectiveness by comparing the triptans used before and after starting a migraine preventive drug as triptans are a specific migraine treatment, but this is still an indirect measure of migraine days. The effect of preventive drugs on the use of non‐specific acute drugs was not assessed. Some migraine preventive drugs, including flunarizine, were not included as these were either not approved for use and/or reimbursement in Norway during the study period. CGRPabs were introduced in Norway in 2018 and approved for reimbursement for chronic migraine in December 2019. Reimbursement beyond the first 90 days of treatment is conditional on proof that the patient experienced at least a 30% reduction in moderate to strong migraine headaches, therefore, the estimation of effectiveness for these drugs beyond the initial 90 days should be interpreted with caution.

This study included all migraine preventive drugs regardless of the indication for use, the rationale being that if a drug has migraine prophylactic properties it will reduce migraine days in migraineurs. This practice is supported by the response rate to beta blockers in our study, and is in line with previous literature [6]. Oral migraine preventive drugs are often used for several indications [7]. In contrast, the parenteral drugs BtA and CGRPabs in the present study were only used for migraine. This difference may have understated the effectiveness of oral drugs in comparison to the parenteral options, as the dose may have been titrated to treat the non‐migraine indication instead of the migraine, and their use depends on patient adherence. As patients may have not expected an effect on migraine, the placebo effect would impact the outcomes less for these drugs. On the other hand, using the migraine preventive drug for non‐migraine indications could have prolonged the duration of use despite lack of effect on migraine, increasing retention. Finally, as we wanted to assess the retention and effectiveness in the complete population, we did not stratify analyses according to age groups but instead adjusted for age.

In conclusion, responder rates to the different drugs ranged from 30% to 50%, and there were large differences in retention and effectiveness among the drugs. All drug groups and drugs studied reduced triptan prescription fills. Overall, the results showed favorable response of CGRPabs, amitriptyline, and simvastatin over beta blockers, while topiramate and clonidine were associated with poorer outcome.

## AUTHOR CONTRIBUTIONS

All authors contributed to the design of the study and the choice of research questions. Marte H. Bjørk: Study design, interpretation of results, preparation of the manuscript, and approval of the manuscript. Solveig Borkenhagen, Francisco Oteiza, Erik Magnus Sæther and Christoffer Bugge: Data collection, study design, analysis, interpretation of results, preparation of the manuscript, and approval of the manuscript. Aud N. Dueland and Frank E. Sørgaard: Study design, interpretation of results, and approval of the manuscript.

## FUNDING INFORMATION

This study was funded by Novartis Norge AS.

## CONFLICT OF INTEREST STATEMENT

Dr. Bjørk reports receiving speakers honoraria from Teva, Eisai and Lilly, advisory board honoraria from Jazz Pharmaceuticals, Angelini Pharma, Lundbeck, Pfizer and Eisai, consultancy honoraria from Novartis, and institutional grants from Sanofi. Aud N. Dueland reports receiving speakers honoraria from AbbVie, Lilly, Lundbeck, Novartis, Roche and Teva, advisory board honoraria from Lilly, Lundbeck, Pfizer and Teva, and consultancy honoraria from AbbVie and Novartis. Frank E. Sørgaard is a medical advisor at Novartis Norway AS. Erik Magnus Sæther, Christoffer Bugge, Francisco Oteiza and Solveig Borkenhagen are employed by Oslo Economics, which provides consulting services in health economics to both private and public healthcare providers. Oslo Economics received funding from Novartis Norway AS to conduct the current study.

## Supporting information


Data S1:

## Data Availability

The data that support the findings of this study are available from Norwegian Institute of Public Health. Restrictions apply to the availability of these data, which were used under license for this study. Data are available from the Norwegian Institute of Public Health upon application.
