# Supplementary material for: Comparative retention and effectiveness of migraine preventive treatments: A nationwide registry‐based cohort study
Source: Eur J Neurol. 2023 Sep 27;31(1):e16062. doi: 10.1111/ene.16062 (PMC11235668; doi:10.1111/ene.16062)
Supplement: Supplementary file 1 — Data S1: [file ENE-31-e16062-s001.docx]

**Supplement to “Comparative retention and effectiveness of migraine preventive treatment: a nationwide registry-based cohort study”**

**Table of Contents**

[eMethods 2](#_Toc113437579)

[Additional Figures and Tables 3](#_Toc113437580)

[eFigure 1: Sample Selection 3](#_Toc113437581)

[eTable 1: Migraine preventive medicines and the minimum daily dose assumed 4](#_Toc113437582)

[eTable 2: Triptans used as indicators for migraine patients and their defined daily dose (DDD) 4](#_Toc113437583)

[eTable 3: Covariates used in retention analysis (a) and relevant comorbidities in the analysis of migraine patients selected for analysis (b) 5](#_Toc113437584)

[eFigure 2: Balance check from propensity score analysis 6](#_Toc113437585)

[Sensitivity analyses 8](#_Toc113437586)

[eTable 4: Relative effectiveness results for patients stratified by triptan frequency (DDD). 8](#_Toc113437587)

[eFigure 3: Reductions in triptan use after extending the baseline period for BtA by 90 days 9](#_Toc113437588)

[eFigure 4: Effectiveness (>30 % reduction) for patients with migraine indicator and monotherapy 10](#_Toc113437589)

[References 12](#_Toc113437590)

eMethods

We estimated propensity scores for each patient using logistic regression using the covariates included in the main analyses, namely patients age, county of residence at treatment start, number of relevant comorbidities, year of treatment start, previous or simultaneous use of migraine preventive drugs, and amount of triptan DDDs prescribed per month (above or below 16 DDDs within a 30-days period). For each migraine preventive drug separately, patients on the migraine preventive drug (treatment) were compared to patients on beta blockers (active comparator).

We used inverse probability of treatment weights (IPTW) where treated patients were assigned a weight of 1/PS and non-treated (i.e., those on the active comparator, beta blockers) were assigned a weight 1/(1-PS). The target of the analysis is the average treatment effect (ATE) among the population.^1^ Patients with propensity scores outside the overlapping regions for treated and non-treated were excluded (restriction to the common support).

Additional Figures and Tables

eFigure 1: Sample Selection


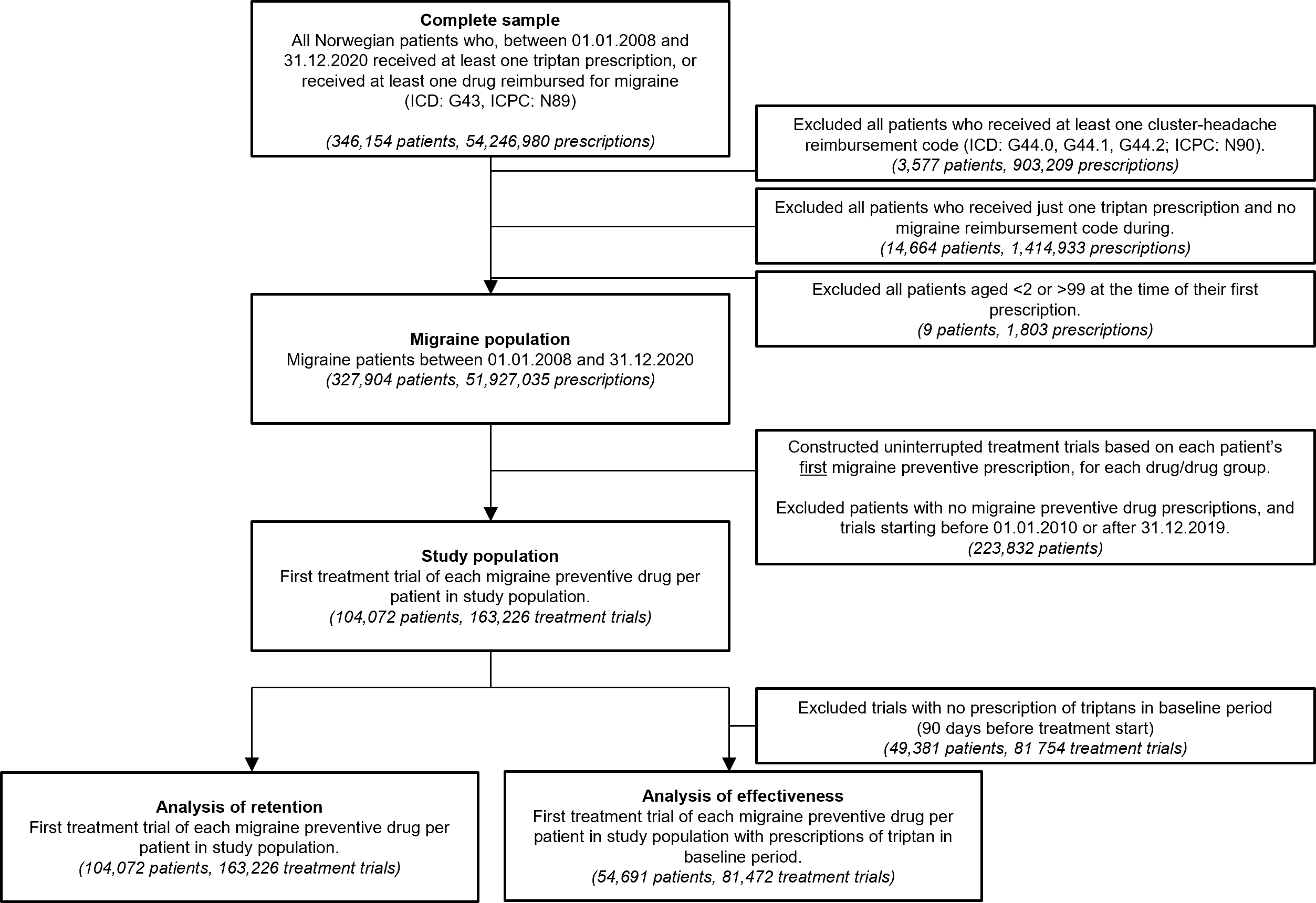


eTable 1: Migraine preventive medicines and the minimum daily dose assumed

| Analysis group | ATC code | Active agent | Minimum effective dose* (mg/day) ^1^ |
| --- | --- | --- | --- |
| Active comparator:  Beta blockers | C07A A05 | Propranolol | 80 |
|  | C07A B02 | Metoprolol | 50 |
| Treatment group 1 | C09A A03 | Lisinopril | 10 |
| Treatment group 2 | C09C A06 | Candesartan | 8 |
| Treatment group 3 | C10A A01 | Simvastatin | 20 |
| Treatment group 4 | N02C X02 | Clonidine | 100 (ug) |
| Treatment group 5:  CGRP-pathway antibodies | N02C D03 | Fremanezumab | 7.39  (225/month) |
|  | N02C D02 | Galcanezumab | 3.94  (120/ month) |
|  | N02C D01 | Erenumab | 2.30  (70/ month) |
| Treatment group 6 | N03A X11 | Topiramate | 50 |
| Treatment group 7 | N06A A09 | Amitriptyline | 20 |
| Treatment group 8 | M03A X01 | Botulinum toxin A | 1.72 U  (155 U/3 months) |

Note: *These are migraine related minimum effective doses based on clinical recommendations in Norway and where non such exist predefined assessments from clinicians with long experience treating migraine patients in Norway (MHB, AND).^2^

eTable 2: Triptans used as indicators for migraine patients and their defined daily dose (DDD)

| ATC-code | Name | DDD | unit | Route of administration |
| --- | --- | --- | --- | --- |
| N02CC01 | Sumatriptan | 20 | mg | Nasal |
|  |  | 50 | mg | Oral |
|  |  | 6 | mg | Parenteral |
| N02CC02 | Naratriptan | 2.5 | mg | Oral |
| N02CC03 | Zolmitriptan | 2.5 | mg | Nasal |
|  |  | 2.5 | mg | Oral |
| N02CC04 | Rizatriptan | 10 | mg | Oral |
| N02CC05 | Almotriptan | 12.5 | mg | Oral |
| N02CC06 | Eletriptan | 40 | mg | Oral |
| N02CC07 | Frovatriptan | 40 | mg | Oral |

Source: WHO Collaborating Centre for Drug Statistics Methodology .^2^

eTable 3: Covariates used in retention analysis (a) and relevant comorbidities in the analysis of migraine patients selected for analysis (b)

| a) Covariates used in retention analysis | | | |
| --- | --- | --- | --- |
| Covariate | Definition | | |
| Age | Age at treatment start (in years) | | |
| Gender | Gender (0=male, 1=female) | | |
| County (at start of treatment) | Agder | | |
|  | Innlandet | | |
|  | Møre og Romsdal | | |
|  | Nordland | | |
|  | Oslo | | |
|  | Rogaland | | |
|  | Troms og Finnmark | | |
|  | Trøndelag | | |
|  | Vestfold og Telemark | | |
|  | Vestland | | |
|  | Viken | | |
| Year | Year of treatment start (2010 as reference year=0) | | |
| Chronic | Ever chronic migraine before treatment period (0=no, 1=yes) | | |
| Other MPDs earlier | Use of other MPDs earlier (number of other MPD groups used before) | | |
| Monotherapy | No other MPDs simultaneously (0=several MPDs, 1=monotherapy) | | |
| Comorbidities | Number of relevant comorbidities before treatment period (see below) | | |
|  |  | | |
| **b) Relevant comorbidities** | | | |
| **Condition** | | **ICD-10 codes** | **ICPC codes** |
| Acute myocardial infarction | | I21, I22, I252 | K75 |
| Congestive heart failure | | I50 | K77 |
| Hypertension | | I10-I16 | K86, K87 |
| Hearth rhythm disorders (atrial fibrillation) | | I48 | K78 |
| Renal disease | | N03, N052, N053, N054, N055, N056, N072, N073, N074, N01, N18, N19, N25 | U88, U99 |
| Depression | | F32, F33 | P76 |
| Anxiety disorder | | F40, F41 | P74 |
| Nicotine replacement products (Cyban etc.) | | HZ80, HZ90 |  |
| Epilepsy | | G40 | N88 |
| Mood disorders | | F30, F31, F34, F39 | P73 |
| Type 2 diabetes (proxy for obesity, inactivity) | | E11 | T90 |
| Asthma | | J45, J46 | R96 |

Note: Covariates used in retention analysis and relevant comorbidities in the analysis of migraine patients selected for analysis. In the case of the county variable, each county dummy took a value of 1 if the patient lived there, or zero otherwise. The same is done for comorbidities and other migraine preventive drugs earlier and simultaneously. Age and baseline triptan use are continuous variables, while the others are discrete. MPD: migraine preventive drugs

eFigure 2: Balance check from propensity score analysis

| Lisinopril  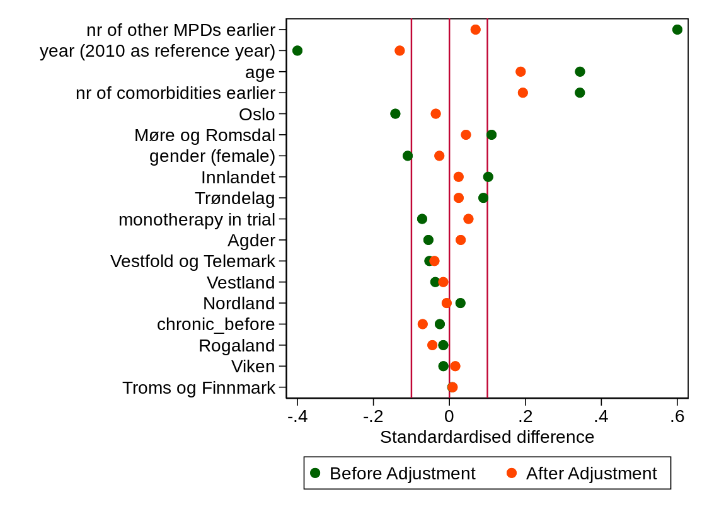 | Candesartan  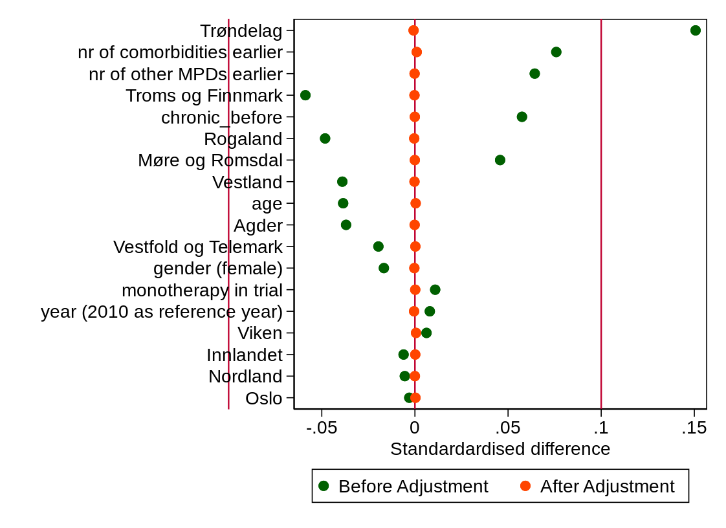 |
| --- | --- |
| **Simvastatin**  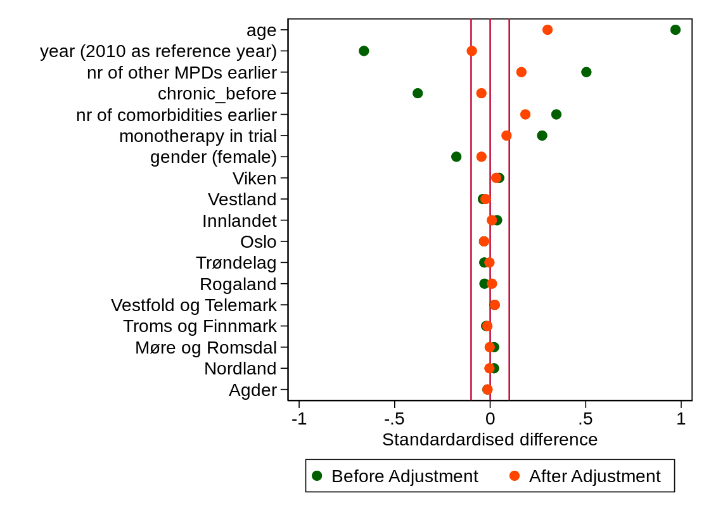 | **Clonidine**  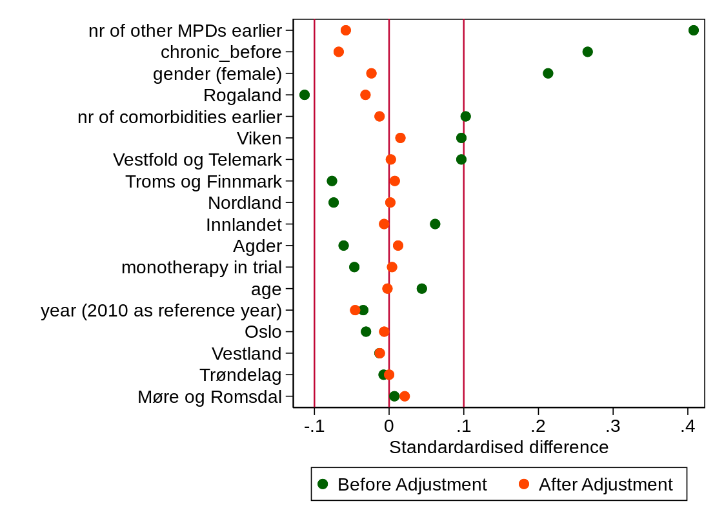 |
| **CGRPabs**  **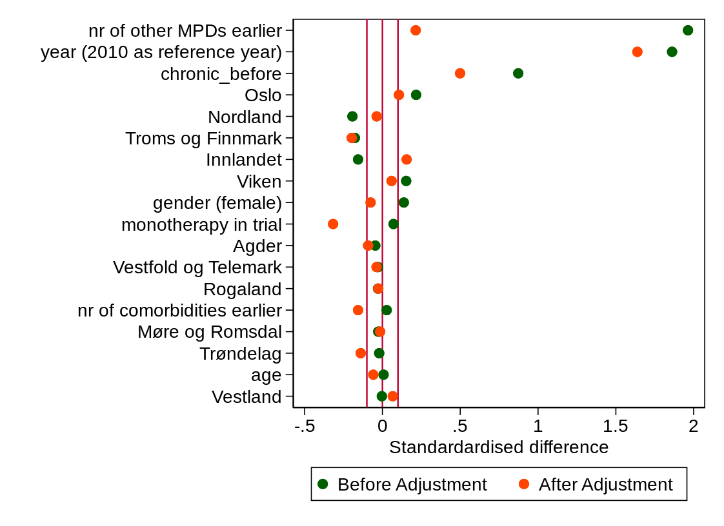** | **Topiramate**  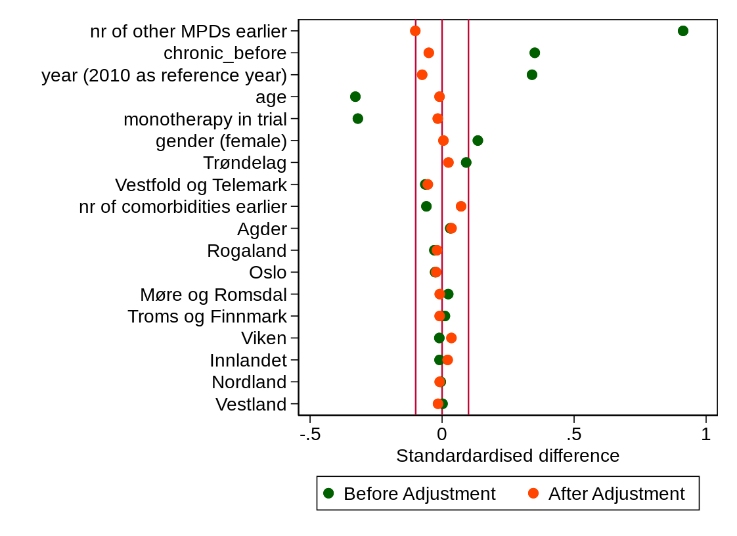 |
| **Amitriptyline**  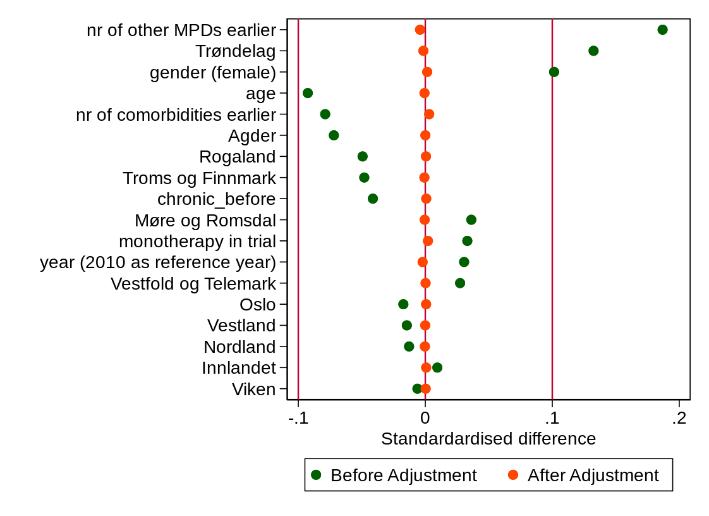 | **BtA**  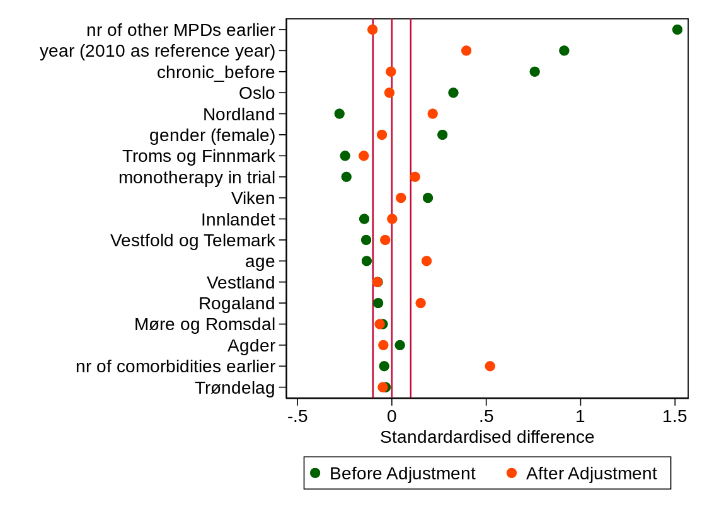 |

Note: Results from balance checks on the relevant covariates before and after propensity score adjustment, by drug group.

Sensitivity analyses

eTable 4: Relative effectiveness results for patients stratified by triptan frequency (DDD).

| Exposure | N total | N with >30 % reduction | Share with 30 % triptan reduction | Crude OR  [95 % CI] | Adjusted OR [95 % CI] | PS adjusted OR [95 % CI] | |
| --- | --- | --- | --- | --- | --- | --- | --- |
| *a) Patients with ≥16 DDD* | | | | | | |  |
| Beta blockers (reference) | 16,581 | 7,650 | 46.14 % |  |  |  | |
|  |  |  |  |  |  |  | |
| Candesartan | 15,021 | 6,761 | 45.01 % | 0.956 | 0.956 | 0.956 | |
|  |  |  |  | [0.91-1.00] | [0.91-1.00] | [0.91-1.00] | |
| Amitriptyline | 11,167 | 5,332 | 47.75 % | 1.067 | 1.113 | 1.11 | |
|  |  |  |  | [1.02-1.12] | [1.06-1.17] | [1.06-1.17] | |
| Simvastatin | 3,128 | 1,535 | 49.07 % | 1.125 | 1.161 | 1.257 | |
|  |  |  |  | [1.04-1.21] | [1.07-1.26] | [1.14-1.39] | |
| Topiramate | 5,086 | 2,190 | 43.06 % | 0.883 | 0.98 | 0.916 | |
|  |  |  |  | [0.83-0.94] | [0.91-1.05] | [0.83-1.01] | |
| Clonidine | 1,924 | 774 | 40.23 % | 0.786 | 0.849 | 0.865 | |
|  |  |  |  | [0.71-0.87] | [0.77-0.94] | [0.78-0.96] | |
| BtA | 2,968 | 1,164 | 39.22 % | 0.753 | 0.899 | 0.698 | |
|  |  |  |  | [0.70-0.82] | [0.82-0.99] | [0.52-0.94] | |
| Lisinopril | 1,063 | 448 | 42.14 % | 0.85 | 0.92 | 0.984 | |
|  |  |  |  | [0.75-0.96] | [0.81-1.05] | [0.84-1.15] | |
| CGRPabs | 1,112 | 603 | 54.23 % | 1.383 | 1.694 | 1.347 | |
|  |  |  |  | [1.22-1.56] | [1.46-1.97] | [0.88-2.07] | |
| *b) Patients with <16 DDD* | | | | | | | |
| Beta blockers | 7,267 | 4,412 | 60.71 % |  |  |  | |
|  |  |  |  |  |  |  | |
| Candesartan | 5,801 | 3,409 | 58.77 % | 0.922 | 0.924 | 0.928 | |
|  |  |  |  | [0.86-0.99] | [0.86-0.99] | [0.86-1.00] | |
| Amitriptyline | 5,349 | 3,469 | 64.85 % | 1.194 | 1.242 | 1.223 | |
|  |  |  |  | [1.11-1.28] | [1.15-1.34] | [1.13-1.32] | |
| Simvastatin | 2,968 | 1,922 | 64.76 % | 1.189 | 1.186 | 1.31 | |
|  |  |  |  | [1.09-1.30] | [1.08-1.31] | [1.18-1.45] | |
| Topiramate | 454 | 280 | 60.00 % | 1.041 | 1.159 | 1.056 | |
|  |  |  |  | [0.86-1.27] | [0.95-1.42] | [0.85-1.31] | |
| Clonidine | 960 | 576 | 61.67 % | 0.971 | 1.034 | 0.986 | |
|  |  |  |  | [0.85-1.11] | [0.89-1.20] | [0.81-1.20] | |
| BtA | 117 | 71 | 60.68 % | 0.999 | 0.789 | 7.677 | |
|  |  |  |  | [0.69-1.45] | [0.53-1.18] | [2.39-24.63] | |
| Lisinopril | 492 | 303 | 61.59 % | 1.037 | 1.017 | 1.043 | |
|  |  |  |  | [0.86-1.25] | [0.84-1.23] | [0.84-1.30] | |

Note: Odds ratios from crude, adjusted and PS adjusted regressions for patients with ≥16 DDD (upper panel) and <16 DDD (lower panel). CGRPabs were excluded from the analysis of patients with <16 DDD due to small sample size.

eFigure 3: Reductions in triptan use after extending the baseline period for BtA by 90 days

**
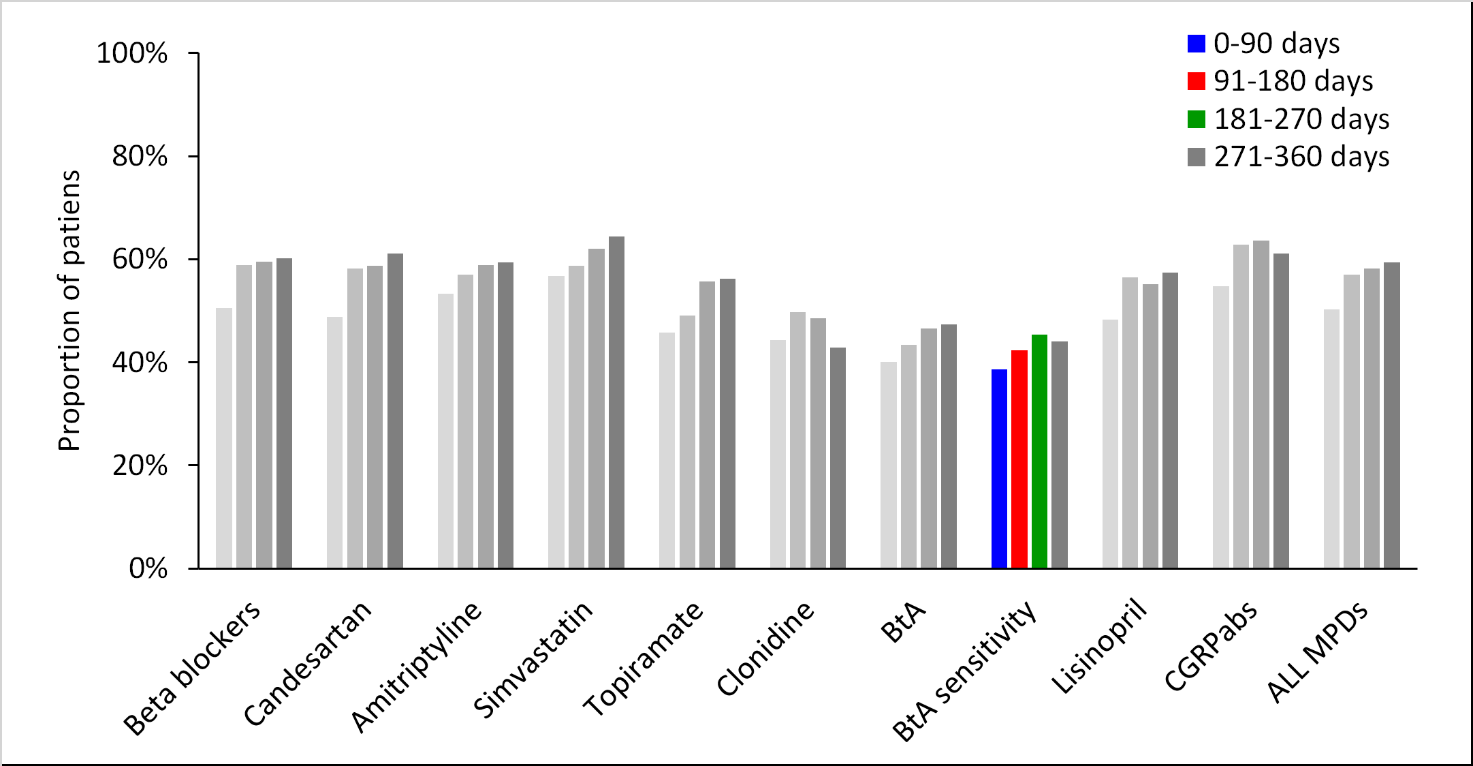
**Note**:** Proportion of patients on preventive migraine therapy with >30 % reduction in triptan use in each observation period, by drug group. Colored bars highlight results when extending the baseline period for BtA by 90 days.

eFigure 4: Effectiveness (>30 % reduction) for patients with migraine indicator and monotherapy


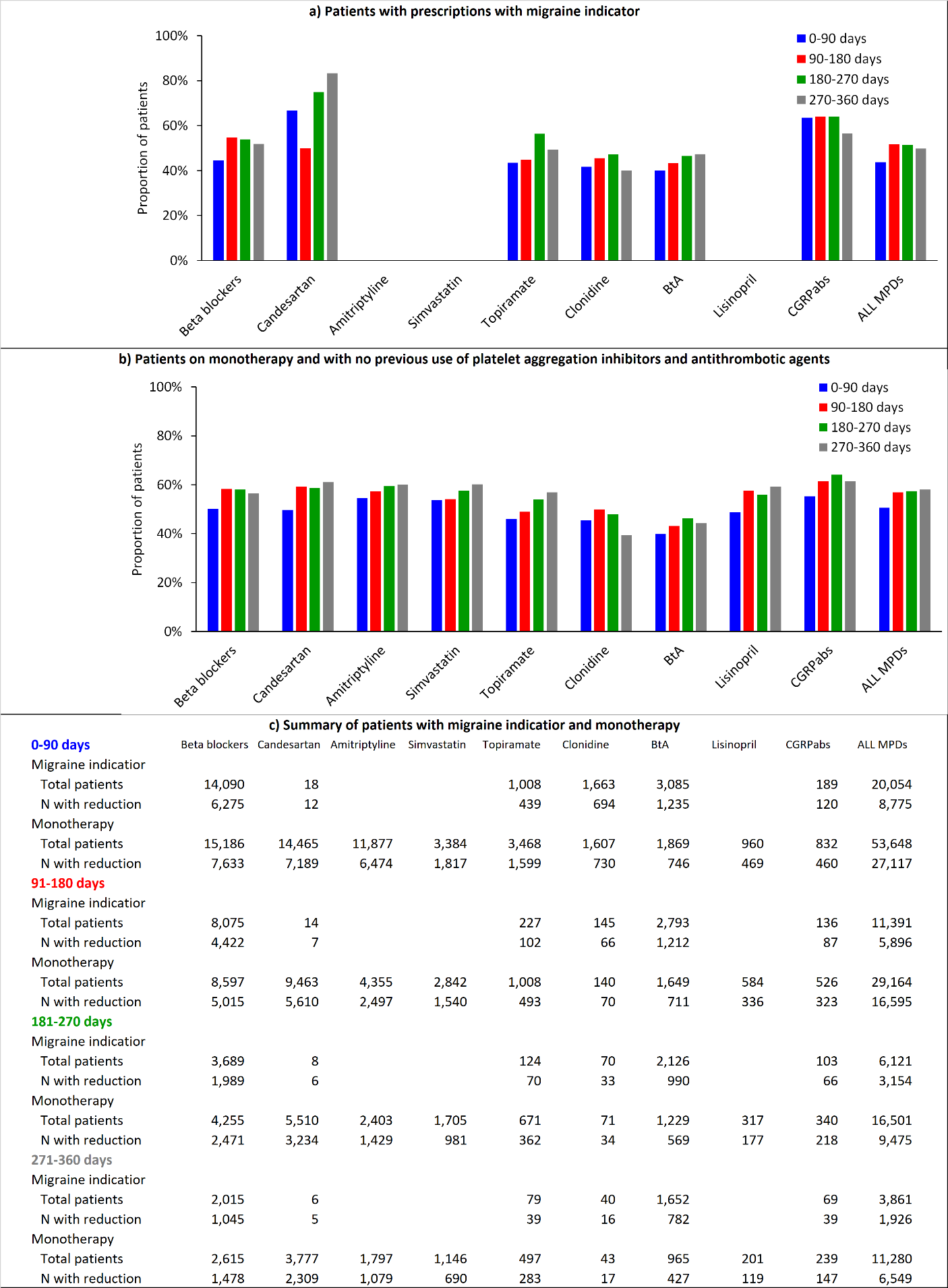

Note: Proportion of patients on preventive migraine therapy with >30 % reduction in triptan use in each observation period, by drug group for two subsamples. Upper panel shows patients with prescriptions containing an explicit migraine reimbursement code only. Lower panel is restricted to patients on a single migraine preventive drug and with no use of platelet aggregation antibodies nor antithrombotic agents.

References

**1.** Desai RJ, Franklin JM. Alternative approaches for confounding adjustment in observational studies using weighting based on the propensity score: a primer for practitioners. *BMJ.* 2019;367:l5657.

**2.** Norwegian Advisory Unit on Headaches. Norwegian neurological guidelines. Migraine*.* Vol 2022: Norwegian Neurological Assosiation; 2022.
